# Estimation of Heart Rate and Respiratory Rate from PPG Signal Using Complementary Ensemble Empirical Mode Decomposition with both Independent Component Analysis and Non-Negative Matrix Factorization

**DOI:** 10.3390/s20113238

**Published:** 2020-06-06

**Authors:** Ruisheng Lei, Bingo Wing-Kuen Ling, Peihua Feng, Jinrong Chen

**Affiliations:** School of Information Engineering, Guangdong University of Technology, Guangzhou 510006, China; xelawk@gmail.com (R.L.); Peihua93@163.com (P.F.); lydmom32@gmail.com (J.C.)

**Keywords:** photoplethysmography, heart rate, respiratory rate, complementary ensemble empirical mode decomposition, mode mixing, independent component analysis, non-negative matrix factorization

## Abstract

This paper proposes a framework combining the complementary ensemble empirical mode decomposition with both the independent component analysis and the non-negative matrix factorization for estimating both the heart rate and the respiratory rate from the photoplethysmography (PPG) signal. After performing the complementary ensemble empirical mode decomposition on the PPG signal, a finite number of intrinsic mode functions are obtained. Then, these intrinsic mode functions are divided into two groups to perform the further analysis via both the independent component analysis and the non-negative matrix factorization. The surrogate cardiac signal related to the heart activity and another surrogate respiratory signal related to the respiratory activity are reconstructed to estimate the heart rate and the respiratory rate, respectively. Finally, different records of signals acquired from the Medical Information Mart for Intensive Care database downloaded from the Physionet Automated Teller Machine (ATM) data bank are employed for demonstrating the outperformance of our proposed method. The results show that our proposed method outperforms both the digital filtering approach and the conventional empirical mode decomposition based methods in terms of reconstructing both the surrogate cardiac signal and the respiratory signal from the PPG signal as well as both achieving the higher accuracy and the higher reliability for estimating both the heart rate and the respiratory rate.

## 1. Introduction

With increasing the life pressure, the cardiorespiratory diseases [1] became the major death reasons of the humans. Thus, it is necessary for the general public to monitor the cardiorespiratory activities [2] so that any abnormal heart situation and any abnormal respiration situation such as the acute physiologic deterioration [3], the cardiovascular diseases [4] and the long term cardiovascular related illnesses [5] can be detected earlier. In addition, both the postoperative treatment [6] and the rehabilitation management [7] can be performed in the early stage. It is worth noting that both the heart rate and the respiratory rate are the important parameters for representing the health conditions. As these signals can be estimated via many consumer electronic devices [8], the general public can monitor the cardiorespiratory activities via these signals with the continuous, noninvasive and comfortable means. However, both the commonly used electrocardiogram (ECG) based heart beat monitoring [9] means and the nasal thermistor based respiratory activity monitoring [10] means are uncomfortable for the patient to use.

The PPG signal is a commonly used signal for measuring the oxygen saturation in the blood. The PPG signal composes of different components. These components are mainly modulated by the heart activities, the respiration activities and other physiological activities [11]. Hence, they are synchronous with both the cardiac rhythm and the respiratory rhythm [12]. By analyzing the PPG signal, the information of the cardiorespiratory activities such as both the heart rate and the respiratory rate could be estimated. Monitoring the cardiorespiratory activities via the PPG signal is a well established noninvasive technique. Comparing with the ECG technique, the hardware implementation cost is much lower. Therefore, the PPG technique is not only used in both the anesthesia and the intensive care units in the hospital, but it is also implemented in a wearable device and used by the general public for monitoring the health condition.

To estimate both the heart rate and the respiratory rate using the PPG signal, the simple digital filtering techniques have been employed [12,13,14]. However, the performance is highly dependent on the cutoff frequency of the filter. Nevertheless, it was empirically selected. Although there are some analytical methods, these methods are very sensitive to the type of the noise. Hence, the result is very poor if the PPG signal is corrupted by a motion artifact. To address this issue, the estimation method based on the time frequency analysis approach [15,16] such as that based on the wavelet transform method [17,18,19,20] was proposed. For example, both the heart rate and the respiratory rate are estimated by the pulse oximeter using the PPG signal. Although this method is less sensitive to both the noise and the motion artifact, these methods require to select more than one parameter such as both the mother wavelet function and the total number of the decomposition level in the filter bank. In practice, these parameters are unknown.

In the recent years, the empirical mode decomposition was proposed [21] as an adaptive tool for processing both the nonstationary and the nonlinear signals. Many signals in the practice can be represented as the sums of their intrinsic mode functions and their residues. These components are localized in the frequency bands with their center frequencies sorted according to their indices. As a result of this nice property, several empirical mode decomposition based methods [22,23,24,25] were developed to decompose the PPG signals for estimating both the heart rates and the respiratory rates. However, due to both the intermittency and the noises corrupted to the PPG signals, the empirical mode decomposition based method may suffer from the occurrence of the mode mixing phenomenon. To address this problem, the ensemble empirical mode decomposition based method is proposed [26]. It is a noise assisted data analysis algorithm which can avoid the occurrence of the mode mixing phenomenon for processing the PPG signals. By applying the ensemble empirical mode decomposition to both the ECG and the PPG signal as well as employing the fusion approach [27], the respiratory rate was estimated using both the second intrinsic mode function and the third intrinsic mode function. However, this method is not robust in terms of applying to other ECG and PPG signals obtained from other subjects or devices. The joint ensemble empirical mode decomposition and the principal component analysis based method was proposed [28] to improve the robustness for estimating both the heart rate and the respiratory rate from the PPG signal by reducing the mode mixing effect. However, it may still yield an inaccurate result if the intrinsic mode functions unrelated to both the heart activity and the respiratory activity are taken into an account.

The complementary ensemble empirical mode decomposition has recently been proposed to avoid the occurrence of the mode mixing phenomenon. Meanwhile, it is found that the reconstruction error can be significantly suppressed [29]. On the other hand, both the independent component analysis [30,31] and the non-negative matrix factorization [32,33] are the powerful techniques for performing the blind source separation. The joint empirical mode decomposition and the independent component analysis based method have been proposed [34] to perform the source separation for the biomedical signal such as the electroencephalogram (EEG) signal. For extracting the fetal ECG signal from a single channel data, the joint empirical mode decomposition and the non-negative matrix factorization based method has also been proposed [35].

The novelty of this paper is to apply the complementary ensemble empirical mode decomposition, the independent component analysis and the non-negative matrix factorization to separate the PPG into two sets of signals as well as the principal component analysis to fuse these two sets of signals to generate two surrogate signals. Here, the surrogate signals refer to the signal components that are used to calculate the corresponding activities. In this paper, these two surrogate signals are the surrogate cardiac signal and the surrogate respiratory signal that are used to calculate the heart rate and the respiratory rate, respectively. It is worth noting that it is not required to select any parameter in the proposed method. Hence, the proposed method is adaptive. The computer numerical simulation results show that our proposed method could achieve the better results in terms of achieving the higher accuracies of both the estimated heart rate and the estimated respiratory rate as well as the more reliable results in terms of achieving the lower variances of the accuracies of both the estimated heart rate and the estimated respiratory rate. These improvements are important because both the accurate and the reliable heart rate as well as both the accurate and the reliable respiratory rate are essential and critical for the medical diagnosis of the cardiorespiratory diseases.

The outline of this paper is as follows. The existing methods for the analysis used in our proposed method are reviewed in Section 2.1. Then, our proposed method for the estimation of both the heart rate and the respiratory rate is presented in Section 2.2. Next, the computer numerical simulation results are shown in Section 3. Finally, a conclusion is drawn in Section 4.

## 2. Proposed Methods

### 2.1. Reviews on the Existing Methods

#### 2.1.1. Complementary Ensemble Empirical Mode Decomposition

The complementary ensemble empirical mode decomposition [29] is an improved version of the ensemble empirical mode decomposition. The main advantage of this method is to reduce the reconstruction error. Unlike the conventional ensemble empirical mode decomposition [26], a pair of uniformly distributed white noises with one positive valued and one negative valued is added to the original signal. It has been shown that the reconstruction error can be significantly suppressed. The detail procedures are described as follows.

Step 1 Let *N* be the total number of the white noises. Denote a set of positive valued white noise sequences as $\left\{n_{i}\left(t\right)\right\}$ and the corresponding negative valued white noise sequences as $\left\{-n_{i}\left(t\right)\right\}$ for *i* = 1, 2, …, *N*. Let S(t) be the original signal. Let *a* be the gain multiplied to the white noises. Add $an_{i}\left(t\right)$ and $-an_{i}\left(t\right)$ to S(t) for *i* = 1, 2, …, *N*. Denote the sequences corrupted by $an_{i}\left(t\right)$ and $-an_{i}\left(t\right)$ as $r_{i}^{+}\left(t\right)$ and $r_{i}^{-}\left(t\right)$ for *i* = 1, 2, …, *N*, respectively. That is:
  (1) $$r_{i}^{+}\left(t\right)=S\left(t\right)+an_{i}\left(t\right)$$
  and
  (2) $$r_{i}^{-}\left(t\right)=S\left(t\right)-an_{i}\left(t\right).$$
Step 2 Decompose both $r_{i}^{+}\left(t\right)$ and $r_{i}^{-}\left(t\right)$ using the empirical mode decomposition. Let $I_{ij}^{+}\left(t\right)$ and $I_{ij}^{-}\left(t\right)$ be the $j^{th}$ intrinsic mode function of $r_{i}^{+}\left(t\right)$ and $r_{i}^{-}\left(t\right)$, respectively.
Step 3 Let $I_{j}\left(t\right)$ be the $j^{th}$ intrinsic mode function of the reconstructed signal. Here, $I_{j}\left(t\right)$ is reconstructed by averaging $I_{ij}^{+}\left(t\right)$ and $I_{ij}^{-}\left(t\right)$ together. That is:
  (3) $$I_{j}\left(t\right)=\frac{1}{2N}\sum_{i=1}^{N}\left(I_{ij}^{+}\left(t\right)+I_{ij}^{-}\left(t\right)\right).$$
Step 4 Let *p* be the total number of the intrinsic mode functions used for the reconstruction. Let r(t) be the residue based on the reconstruction of the signal using these *p* intrinsic mode functions. That is:
  (4) $$S\left(t\right)=\sum_{j=1}^{p}I_{j}\left(t\right)+r\left(t\right).$$

#### 2.1.2. Independent Component Analysis

The independent component analysis is a statistical method and widely used in many signal processing applications such as in both the blind source separation application and the feature extraction application [30]. Let X be the observed mixed signal matrix, S be the instantaneously independent source and *M* be the mixing matrix. Let the dimension of X be m×n. Let both the total number of independent rows and the total number of the independent columns of both M and S be rank. Then, the dimensions of M and S are m×rank and rank×n, respectively. The blind source separation problem is to separate S from X using M. That is:

(5) X=MS.

To solve this problem, a common algorithm called the FastICA was used. It employs the maximum negative entropy as the search direction to find S [31].

#### 2.1.3. Non-Negative Matrix Factorization

The non-negative matrix factorization is a kind of linear representations of the non-negative data [32,33]. It is also applied in many signal processing applications such as in the blind source separation application. Denote the observed non-negative signal matrix as *V*. Let W be the mixed matrix and *H* be the source signal matrix. Let the dimension of *V* be m×n. Let both the total number of independent rows and the total number of the independent columns of both *W* and *H* be rank. Then, the dimensions of W and H are m×rank and rank×n, respectively. The non-negative matrix factorization problem is to approximate *V* as the product of *W* and *H*. That is:

(6) V≈WH.

Let $W_{i,j}$ and $H_{i,j}$ be the elements in the *i*th row and the *j*th column of W and H, respectively. In this paper, the non-negative matrix factorization with the sparseness constraints [36] is considered. In particular, let ${\left|\left|.\right|\right|}_{0}$ be the zero norm operator. Here, it refers to the total number of the nonzero elements in the operand. Let ∈ be the specification on the maximum number of the nonzero elements in W. Then, the non-negative matrix factorization problem is formulated as the sparse constrained optimization problem such that the two norm error ${\left|\left|V-WH\right|\right|}^{2}$ is minimized subject to ${\left|\left|W\right|\right|}_{0}\leq \in$ as well as both $W_{i,j}\geq 0$ and $H_{i,j}\geq 0$ for all i,j. It is found that a better performance can be achieved compared to other basic algorithms.

### 2.2. Our Proposed Framework for Extracting the Cardiorespiratory Activity from the PPG Signal

**Figure 1:**
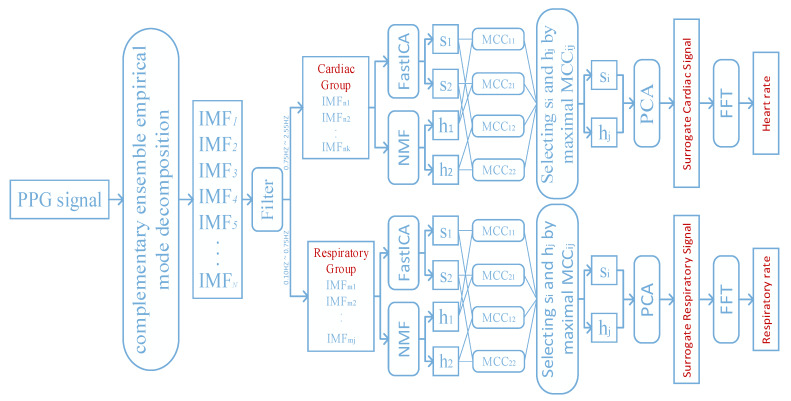
The proposed framework for extracting the cardiorespiratory activity from the PPG signal.

In this paper, we propose a framework for extracting the cardiorespiratory activity from the PPG signal. The framework is shown in Figure 1. It mainly combines the existing methods discussed in Section 2.1. The overall algorithm can be divided into five stages and illustrated as follows.

#### 2.2.1. Decomposition of the PPG Signal Using the Complementary Ensemble Empirical Mode Decomposition

**Figure 2:**
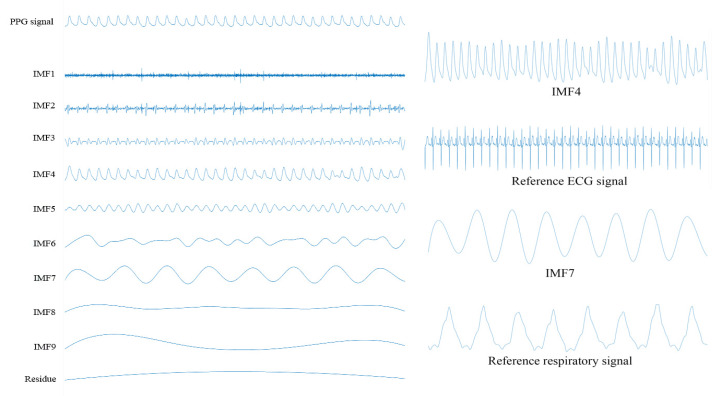
The PPG signal, its intrinsic mode functions as well as both the reference ECG signal and the reference respiratory signal.

It is worth noting that both the estimated heart rate and the estimated respiratory rate are dependent on the sampling rate of the PPG signal. Thus, both the separation of the cardiorespiratory related signals and the extraction of the cardiorespiratory activities from the PPG signal are also dependent on the sampling rate of the PPG signal. In this paper, first a PPG signal is segmented into a finite number of pieces. Here, the duration of each piece of the PPG signal is 30 s. Then, each piece of the PPG signal is decomposed into a finite number of intrinsic mode functions using the complementary ensemble empirical mode decomposition. It is worth noting the total numbers of the intrinsic mode functions of different pieces of the PPG signal may be different. To address this difficulty, the peak frequency of each intrinsic mode function of each piece of the PPG signal is computed. The peak frequencies of the intrinsic mode functions of two consecutive pieces of the PPG signal are linked together by using the dynamical programming approach. The mean and the variance of the peak frequencies of the intrinsic mode functions of each link are computed. It is found that the intrinsic mode functions of the PPG signal in the fourth link and the seventh link are corresponding to the ECG signal and the respiratory signal, respectively. This is because the means of the peak frequencies of the intrinsic mode functions in these two links fall to the ranges of the heart rate and the respiratory rate, respectively. An example of a piece of a PPG signal, its intrinsic mode functions as well as both the reference ECG signal and the reference respiratory signal are shown in Figure 2. It can be seen from the figure that the fourth intrinsic mode function of this piece of the PPG signal is the intrinsic mode function that its frequency is the closest to that of the reference ECG signal. In addition, the seventh intrinsic mode function of this piece of the PPG signal is the intrinsic mode function that its frequency is the closest to that of the reference respiratory signal. In fact, the frequencies of all the intrinsic mode functions in the fourth link and the seventh link for this subject are the closest to those of the reference ECG signal and the reference respiratory signal, respectively. Additionally, similar results are found for all other subjects.

#### 2.2.2. Filtering on the Intrinsic Mode Functions

The normal ranges of the heart rates and the respiratory rates for the young population (including both children between 2 and 18 years old and young adults) are between 45 and 145 beats per minute as well as between 8 and 45 breaths per minute [23,37], respectively. Therefore, this paper chooses the frequency band between 0.1 Hz and 2.55 Hz as the possible signal band. Then, the intrinsic mode functions with the dominating frequencies lying in the range between 0.75 Hz and 2.55 Hz are selected to form a group of the candidate cardiac intrinsic mode functions. On the other hand, the intrinsic mode functions with the dominating frequency lying in the range between 0.1 Hz and 0.75 Hz are selected as a group of the candidate respiratory modes. Hence, the intrinsic mode functions in these frequency bands are categorized into two groups denoted as the cardiac group and the respiratory group, respectively.

#### 2.2.3. Performing both the Independent Component Analysis and the Non-Negative Matrix Factorization on the Intrinsic Mode Functions

To separate the source signals embedding in each group, the set of intrinsic mode functions in the same group are represented as a matrix X with *m* rows and *n* columns. Here, each column of X is an intrinsic mode function. For performing the non-negative matrix factorization, the observed matrix is required to be non-negative. Hence, by letting each column of X subtracted from its minimum as the column of a non-negative matrix V, the non-negative matrix is obtained. Now, the source signal separation problem is formulated as two optimization problems defined in Equations (5) and (6), respectively. Then, by applying the FastICA and the non-negative matrix factorization with the sparseness constraints to *X* and *V*, respectively, these two rank×n source signal matrices S and H as well as these two m×rank mixed matrices M and W are found. In this paper, rank is set to 2 to separate four source signals. Then, they are mapped to the range between −1 and 1. Finally, they are denoted as $s_{1}$, $s_{2}$, $h_{1}$ and $h_{2}$. After the source signals $s_{1}$, $s_{2}$, $h_{1}$ and $h_{2}$ are obtained from each group, the maximal cross correlation coefficient (MCC) is employed to select a pair of source signals to form a surrogate signal. Let $R_{ij}$ be the cross correlation function between the source signal $s_{i}$ and $h_{j}$. Here, the MCC between $s_{i}$ and $h_{j}$ is defined as

(7) $$\mathrm{MCC}=maximum\left(\left|R_{ij}\right|\right).$$

Obviously, the MCC can clearly indicate the similarity between the source signals obtained by different algorithms without affected by the phase difference between these source signals. Therefore, this paper selects a pair of source signal $s_{i}$ and $h_{j}$ corresponding to the maximal MCC as the surrogate signals.

#### 2.2.4. Performing the Principal Component Analysis on the Selected Pairs of Source Signals

In order to both fuse a pair of the selected source signals and retain most of their variations, the first principal components obtained by applying the principal component analysis on the pairs of the source signals from a cardiac group and the respiratory group were used as a surrogate cardiac signal and the respiratory signal, respectively. Therefore, the obtained surrogate cardiac signal and the respiratory signal indicate the cardiac activity and the respiratory activity, respectively.

#### 2.2.5. Estimation of Both the Heart Rate and the Respiratory Rate Using the Surrogate Signals

In order to extract the cardiorespiratory activities such as both the heart rate and the respiratory rate from the corresponding surrogate signals, the FFTs of these surrogate signals are computed. Let $f_{HR}$ and $f_{RR}$ be the heart rate frequency and the respiratory rate frequency, respectively. They are defined as the peak frequencies of the surrogate heart signal and the surrogate respiratory signal, respectively. Let HR and RR be the estimated heart rate and the estimated respiratory rate, respectively. That is:

(8) $$HR=f_{HR}*60\;(\mathrm{beats}/\min)$$

and

(9) $$RR=f_{RR}*60\;(\mathrm{breaths}/\min),$$

respectively.

## 3. Computer Numerical Simulation Results

### 3.1. Database

The Medical Information Mart for Intensive Care database [38] contains 720 complete records of 90 different intensive care unit patients. Every record contains the signals such as the ECG signals, the PPG signals and the respiratory signals with 10 min duration. These signals are acquired simultaneously and sampled at 125 Hz. In this paper, all these 720 records are used to estimate both the heart rate and the respiratory rate. In addition, the obtained results are compared to the reference manually computed heart rate and the reference manually computed respiratory rate, respectively. However, due to the page limits, only four records of four subjects with the subject identification numbers 055m, 212m, 220m and 408m were demonstrated below. These four records are demonstrated because they correspond to different diseases. In particular, 055m is suffered from the respiratory failure, 212m is suffered from the pulmonary edema, 220m is suffered from the brain injury and 408m is suffered from the bleeding. In fact, other records also exhibit the similar results.

### 3.2. Performances

**Figure 3:**
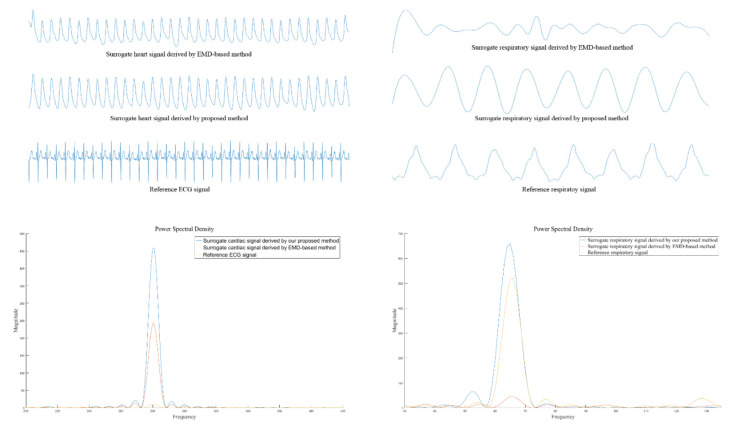
Both the surrogate cardiac signal and the surrogate respiratory signal obtained by both the empirical mode decomposition based method [23] and our proposed method as well as both the reference ECG signal and the reference respiratory signal.

Figure 3 shows both the surrogate cardiac signal and the surrogate respiratory signal obtained via both the empirical mode decomposition based method [23] and our proposed method. In addition, both the reference ECG signal and the reference respiratory signal are shown in Figure 3. From the figure, it can be seen that the rhythms of both the cardiac activity and the respiratory activity obtained by our proposed method are more obvious compared to those obtained by the empirical mode decomposition based method. This is particularly obvious for the surrogate respiratory signal. In addition, the artifact effect such as the boundary effect obtained by our proposed method is less obvious than that obtained by the empirical mode decomposition based method.

To quantitatively evaluate the performance of our proposed method, both our proposed method and the empirical mode decomposition based method are applied to the signals with the lengths equal to 30 s durations. Here, the signals with only 30 s durations are used for evaluating the numerical performances. This is because it can save the required computational powers. Let $\tilde{HR_{i}}$ be the estimated heart rate, $HR_{i}$ be the manually computed heart rate, $\tilde{RR_{i}}$ be the estimated respiratory rate, $RR_{i}$ be the manually computed respiratory rate and *N* be the total number of the samples of the entire record within the 30 s duration. Let $ACC_{HR}$ and $ACC_{RR}$ be the accuracy of the estimated heart rate and the accuracy of the estimated respiratory rate, respectively. More precisely, they are defined as

(10) $$ACC_{HR}=\frac{1}{N}\sum_{i=1}^{N}\left(1-\frac{\left|\tilde{HR_{i}}-HR_{i}\right|}{HR_{i}}\right)\times 100\%$$

and

(11) $$ACC_{RR}=\frac{1}{N}\sum_{i=1}^{N}\left(1-\frac{\left|\tilde{RR_{i}}-RR_{i}\right|}{RR_{i}}\right)\times 100\%.$$

The results were shown in Table 1. It can be seen from Table 1 that the digital filtering approach achieves the lowest accuracies on the estimations of both the heart rate and the respiratory rate. On the other hand, the empirical mode decomposition based method achieves the similar accuracy on the estimation of the heart rate compared to that of our proposed method. However, the empirical mode decomposition based method achieves a lower accuracy on the estimation of the respiratory rate compared to that based on our proposed method. In particular, the $ACC_{RR}$ of the subjects with the subject numbers 055m and 220m achieved by our proposed method are 7.94% and 10.73% higher than those achieved by the empirical mode decomposition based method, respectively. These are the significant results. Overall, both the means and the variances of both the $ACC_{HR}$ and the $ACC_{RR}$ over all these 90 subjects achieved by our proposed method, the empirical mode decomposition based method and the digital filtering approach are computed and listed in Table 2. From here, it can be seen that our proposed method achieves the highest average accuracies compared to both the digital filtering approach and the empirical mode decomposition based method. In addition, our proposed method is more reliable compared to both the digital filtering approach and the empirical mode decomposition based method.

**Figure 4:**
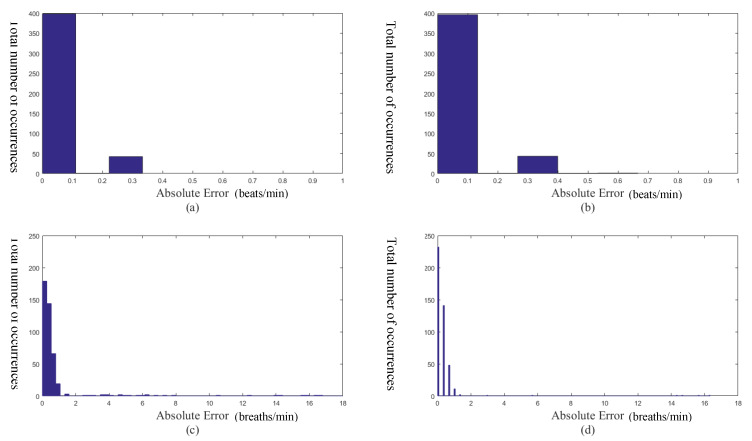
(**a**) The absolute error of the heart rate obtained by the empirical mode decomposition based method. (**b**) The absolute error of the heart rate obtained by our proposed method. (**c**) The absolute error of the respiratory rate obtained by the empirical mode decomposition based method. (**d**) The absolute error of the respiratory rate obtained by our proposed method.

Figure 4a,b show the histograms of the absolute errors of the estimated heart rates obtained by the empirical mode decomposition based method and our proposed method, respectively. Figure 4c,d show the histograms of the absolute errors of the estimated respiratory rates obtained by the empirical mode decomposition based method and our proposed method, respectively. Let $AE_{HR}$ and $AE_{RR}$ be the absolute error of the estimated heart rate and the absolute error of the estimated respiratory rate, respectively. That is:

(12) $$AE_{HR}=\left|\tilde{HR_{i}}-HR_{i}\right|$$

and

(13) $$AE_{RR}=\left|\tilde{RR_{i}}-RR_{i}\right|,$$

respectively. It can be seen From Figure 4a,b that the histogram of the absolute errors of the estimated heart rates obtained by the empirical mode decomposition based method is similar to that obtained by our proposed method. On the other hand, it can be seen from Figure 4c,d that there are some large values of $AE_{RR}$ based on the empirical mode decomposition based method (there are some bars in the histogram with small occurrences at the large absolute errors). Whereas, there is no large value of $AE_{RR}$ based on our proposed method. Therefore, our proposed method is more reliable than the empirical mode decomposition based method for computing the surrogate respiratory signal.

**Figure 5:**
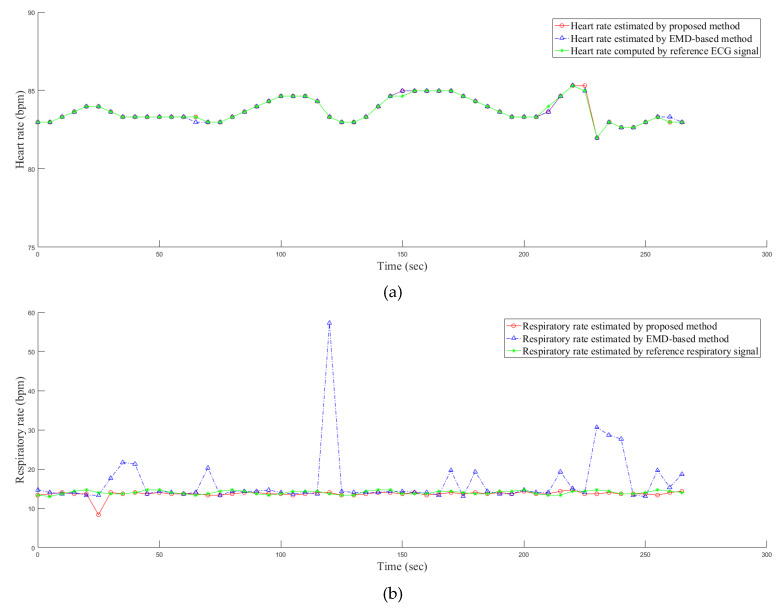
The estimation of (**a**) the heart rate and (**b**) the respiratory rate of a PPG signal within the 270 s duration.

The results on the estimation of both the heart rate and the respiratory rate of a PPG signal within the 270 s duration are shown in the Figure 5. Here, more than 30 s are shown because the signals with the longer durations can show the parts of the surrogate cardiac signal with the variations such as the durations between 110 s and 150 s as well as those between 200 s and 230 s in the surrogate cardiac signal. From the figure, it can be seen that our proposed method outperforms the empirical mode decomposition based method particularly for the surrogate respiratory signal.

Finally, the required computational power of the proposed method is evaluated. An Intel(R) Xeon(R) E3-1225 V2 CPU operating at 3.2 GHz with a 16 GB memory is employed for performing the computer numerical simulations. All the algorithms are executed using the Matlab Version 7.11.0.584 (R2010b) operating under the 64 bit Microsoft Windows 7 Version 6.1 with Service Pack 1 and Java 1.6.0_17-b04. It is found that the required computational time for processing a signal with the 30 s duration based on our proposed method is 0.12 s, which is acceptable in most real time applications.

## 4. Conclusions

This paper applies the complementary ensemble empirical mode decomposition, the independent component analysis and the non-negative matrix factorization to separate the PPG into two sets of signals as well as the principal component analysis to fuse these two sets of signals to generate two surrogate signals. These two surrogate signals are used to calculate the heart rate and the respiratory rate, respectively. The computer numerical simulation results show that our proposed method could achieve both the more reliable and the more accurate results compared to both the digital filtering approach and the conventional empirical mode decomposition based method.

## Figures and Tables

**Table 1:** The $ACC_{HR}$ and the $ACC_{RR}$ of the four subjects obtained by our proposed method, the empirical mode decomposition based method and the digital filtering approach.

Methods	055m		212m		220m		408m	
	$ACC_{HR}$	$ACC_{RR}$	$ACC_{HR}$	$ACC_{RR}$	$ACC_{HR}$	$ACC_{RR}$	$ACC_{HR}$	$ACC_{RR}$
Our proposed method	99.96%	95.95%	99.97%	99.82%	99.90%	96.54%	99.96%	98.79%
Empirical mode decomposition based method [23]	99.96%	88.01%	99.98%	98.79%	99.93%	85.81%	99.85%	95.02%
Digital filtering approach [13]	92.34%	87.41%	92.41%	88.12%	91.78%	84.19%	92.31%	84.24%

**Table 2:** Both the means and the variances of both the $ACC_{HR}$ and the $ACC_{RR}$ over all these 90 subjects achieved by our proposed method, the empirical mode decomposition based method and the digital filtering approach.

Methods	Means		Variance	
	$ACC_{HR}$	$ACC_{RR}$	$ACC_{HR}$	$ACC_{RR}$
Our proposed method	99.95%	97.78%	0.0010%	3.3560%
Empirical mode decomposition based method [23]	99.93%	91.91%	0.0033%	36.4755%
Digital filtering approach [13]	92.01%	85.12%	2.41%	25.12%
